# Loss of Pancreas upon Activated Wnt Signaling Is Concomitant with Emergence of Gastrointestinal Identity

**DOI:** 10.1371/journal.pone.0164714

**Published:** 2016-10-13

**Authors:** Jose Luis Muñoz-Bravo, Alvaro Flores-Martínez, Griselda Herrero-Martin, Sapna Puri, Makoto Mark Taketo, Anabel Rojas, Matthias Hebrok, David A. Cano

**Affiliations:** 1 Unidad de Gestión Clínica de Endocrinología y Nutrición, Hospital Universitario Virgen del Rocío, Sevilla, Spain; 2 Instituto de Biomedicina de Sevilla (IBiS), Hospital Universitario Virgen del Rocío/Consejo Superior de Investigaciones Científicas/Universidad de Sevilla, Sevilla, Spain; 3 Diabetes Center, Department of Medicine, University of California San Francisco, San Francisco, United States of America; 4 Department of Pharmacology, Graduate School of Medicine, Kyoto University, Kyoto, Japan; 5 Centro Andaluz de Biología Molecular y Medicina Regenerativa (CABIMER), Sevilla, Spain; 6 Centro de Investigación Biomédica en Red de Diabetes y Enfermedades Metabólicas Asociadas (CIBERDEM), Madrid, Spain; Western University, CANADA

## Abstract

Organ formation is achieved through the complex interplay between signaling pathways and transcriptional cascades. The canonical Wnt signaling pathway plays multiple roles during embryonic development including patterning, proliferation and differentiation in distinct tissues. Previous studies have established the importance of this pathway at multiple stages of pancreas formation as well as in postnatal organ function and homeostasis. In mice, gain-of-function experiments have demonstrated that activation of the canonical Wnt pathway results in pancreatic hypoplasia, a phenomenon whose underlying mechanisms remains to be elucidated. Here, we show that ectopic activation of epithelial canonical Wnt signaling causes aberrant induction of gastric and intestinal markers both in the pancreatic epithelium and mesenchyme, leading to the development of gut-like features. Furthermore, we provide evidence that β -catenin-induced impairment of pancreas formation depends on Hedgehog signaling. Together, our data emphasize the developmental plasticity of pancreatic progenitors and further underscore the key role of precise regulation of signaling pathways to maintain appropriate organ boundaries.

## Introduction

Organ formation during embryonic development is controlled by a relatively small number of signaling pathways. These pathways, however, need to be precisely regulated in a spatio-temporal manner for the correct development of tissues and organs. Improper activation (or inhibition) of these pathways can result in severe impairment of cell specification and differentiation. The pancreas derives from the endodermal germ layer during gastrulation. In mice, the pancreas develops from two independent primordia (dorsal and ventral) of the foregut epithelium around 8.5–9 days of gestation (E8.5–9). The pancreatic epithelium expands and branches into the surrounding mesenchyme by E11.5. This early pancreatic epithelium contains multipotent pancreatic progenitor cells that give rise to all adult pancreatic cell types. Around E13.5, two distinct cellular domains are formed in the pancreatic epithelium. The epithelial "tips" of the branching epithelium become acinar progenitor cells while the "trunk" domain contains cells that will give rise to endocrine and ductal cells. This period is known as the "secondary transition", in which extensive endocrine and exocrine cell differentiation takes place. After the secondary transition, the differentiated pancreatic cells undergo further growth and differentiation with endocrine cells organizing into islets of Langerhans. The development of the mammalian pancreas has been intensively studied with the goal of generating ß-cells in vitro for transplantation therapy, significantly advancing our understanding of the underlying molecular mechanisms during the last several decades (see [1–4] for recent reviews on the subject).

The pancreas is of great medical relevance due to the devastating diseases associated with failure of this organ, including diabetes, pancreatitis and pancreatic cancer. Much of the molecular knowledge gained in pancreas development has also contributed to shed light on the mechanisms underlying adult organ homeostasis and metaplasia. Thus, it has become increasingly evident that many of the embryonic signaling pathways involved in pancreas development such as Hedgehog (Hh), Notch and Wnt signaling also play crucial roles in pancreas regeneration and cancer in the adult organism [5–8]. However, less is known about the link between these embryonic signaling pathways and developmental anomalies of the pancreas in humans. The study of these rare conditions in humans has revealed mutations in genes encoding master transcription factors that regulate pancreas embryonic formation in mice (reviewed in [1, 9]) but the role of signaling pathways remains largely unexplored.

Previous studies have established the importance of the canonical Wnt signaling pathway in embryonic pancreas formation as well as in postnatal organ function and homeostasis [10–13]. These studies have revealed a complex role of the canonical Wnt pathway on pancreas development fulfilling different functions at distinct stages of embryonic pancreas development. This is best illustrated by gain-of-function studies that have illuminated the importance of the timing of canonical Wnt activation during pancreas development. Precocious activation of β-catenin in the early stages of pancreas formation in mice (between embryonic day E10.5-E12.5) results in pancreas hypoplasia, while activation at a later time point during embryogenesis causes a marked increase in pancreas organ size [11, 14]. Similarly, overexpression of Wnt ligands in the early pancreatic domain causes severe pancreatic hypoplasia [13, 15]. While these studies indicate an inhibitory role of the canonical Wnt signaling pathway during early stages of pancreas formation, the underlying mechanisms remain to be elucidated. Also, while canonical Wnt signaling pathway seems to be important for the specification and patterning of several endoderm-derived organs such as intestine [16] and lung [17], whether this pathway plays a role in the initial specification and morphogenesis of the pancreas remains unclear.

In the present study, we show that activation of β-catenin in the prepancreatic endoderm impairs pancreas formation. Moreover, we demonstrate that ectopic β-catenin activation can redirect the developmental fate of pancreatic progenitors towards a gastrointestinal identity, a process mediated by Hedgehog signaling.

## Materials and Methods

### Mice

All procedures involving experimental animals were performed in accordance with European and local animal welfare laws, guidelines and policies. All experimental protocols were approved by the IBiS-Virgen del Rocio Ethics Committee and by the Committee on Animal Research at the University of California, San Francisco (permit number AN088473-01C). Animals were euthanized by cervical dislocation.

*Pdx1-Cre* [18], *Rfx6-Cre* [19] and *Ctnnb1*^*tm1Mmt/+*^ [20] mice carrying the floxed exon 3 allele of β-catenin have been previously described. Noon of the day when vaginal plugs are detected is treated as E0.5 day post coitum.

### Histological analyses

Tissue processing, histological and immunohistochemical analyses were performed as previously described [21]. Immunoperoxidase staining was performed with the ABC Elite and DAB kits (Vector). For Alcian Blue staining, deparaffinized slides were incubated in a solution of 10g/l 8GX Alcian blue pH 2.5 for 30 min, washed and counterstained with nuclear fast red (NFR). For Periodic acid-Schiff staining (PAS), deparaffinized slides were incubated in 0.5% periodic acid solution for 5 min at room temperature, rinsed, placed in Schiff reagent (Sigma-Aldrich) for 15 min and counterstained with hematoxylin. For Gomori's one-step trichrome staining, deparaffinized slides were pretreated with Bouin's solution (Sigma-Aldrich) for 1 hour at 56°C, placed in Weigert's iron hematoxylin for 10 min, stained with Gomori trichrome stain (Sigma-Aldrich) for 15 min and differentiated in 0.5% acetic acid for 2 minutes. Fluorescent and bright-field images were captured using a BX-61 microscope (Olympus) or LTC SP2 confocal microscope (Leica). Primary antibodies used are listed in S1 Table. All photomicrographs shown are representative of at least three independent samples of the indicated genotype. To quantify Cpa1-positive area at E13.5, the entire pancreatic tissue was sectioned in 5 μm thick sections and every fifth section was immunostained for Cpa1. The total pancreatic area, identified by morphology and localization, were measured using ImageJ software. For double Cpa1 and E-cadherin immunofluorescence, Cpa1-positive area was expressed as a ratio of total epithelial E-cadherin area to visualize epithelial area.

### Pancreas explants

E10.5 pancreatic rudiments and guts were dissected together and cultured in the air-liquid interface for 3 days on Millicell (Millipore) inserts as previously reported [22]. Briefly, guts were harvested in Hank’s balanced salts solution (HBSS) and cultured in 24-well plates with 350 μl of RPMI medium supplied with 10% fetal bovine serum, containing 100 U/ml penicillin, 100 μg/ml streptomycin and 2mM of L-glutamine. Cyclopamine (C4116 Sigma-Aldrich) was dissolved in DMSO (stock solution 10 mM) and added to the culture at a final concentration of 10 μM as previously described [23]. Medium was changed every other day.

### Whole Mount Immunostaining

Cultured explants were permeabilized and cleared as previously reported [24] with the following modifications. Cultured explants were fixed for 30 min at 4°C in 4% (wt/vol) paraformaldehyde in phosphate-buffered saline (PBS). Primary and secondary antibodies were incubated at 4°C for 24 and 48 hours respectively with gentle rocking. *z-*stack confocal images were taken every 1 μm using a LTC SP2 confocal microscope (Leica). Area quantification was performed with ImageJ (NIH).

### Quantitative real-time PCR

For RNA isolation, dorsal pancreatic buds at different stages were quickly dissected in cold PBS and placed into 10 volumes (w/v) of RNAlater (Qiagen) at 4°C over-night. Total RNA was isolated using the RNeasy Kit (Qiagen) following manufacturer instructions. DNAse I treatment (Sigma) was used prior to retrotranscriptase reaction to ensure elimination of all genomic DNA. cDNA was synthesized using Omniscript reverse transcriptase (Qiagen). Real-time quantitative PCR was performed with SYBR Green PCR Master Mix (Applied Biosystems) using a 7900HT real-time PCR system (Applied Biosystems). Relative quantification of RNA levels was calculated using ΔΔCt method. Cyclophilin A (peptidylprolyl isomerase A—Mouse Genome Informatics) was selected from a panel of 6 reference genes as the most stable. Results are expressed as fold relative to levels in control pancreata (value of 1). Primers were selected and validated for gut and pancreatic tissue in accordance to MIQE guidelines [25]. Primer sequences are listed in S2 Table.

### Microarray Analysis

Pancreata were dissected from E13.5 embryos and stored in RNAlater (Qiagen) until genotyped was performed. Once genotyped, 3 pancreata from *Pdx1-Cre; Ctnnb1*^*tm1Mmt/+*^ and control embryos were pooled and total RNA was isolated using the RNeasy Kit (Qiagen) following manufacturer instructions. Expression analysis was performed in three independent pools of both *Pdx1-Cre; Ctnnb1*^*tm1Mmt/+*^ and control pancreata using Affymetrix Mouse Gene 1.0 ST Array (Affymetrix, Santa Clara, CA) following manufacturer's instructions at CABIMER Genomic Core Facility. Statistical analysis of the microarray data was done by LIMMA (Linear Models for Microarray Analysis) using R software. This analysis applies a t-test empirically adjusted by a Bayes test. Microarray data were deposited in Gene Expression Omnibus database (GEO; accession number: GSE82342).

### Statistical analysis

Significance was determined using two-tailed Student’s *t*-test, and one-way ANOVA (post-hoc Tukey HSD test). *p*<0.05 was considered significant. Data are presented as mean ± s.e.m.

## Results

### Activation of Wnt/ β-catenin induces loss of pancreatic identity

To evaluate the effect of canonical Wnt pathway activation on pancreas formation, we crossed mice carrying a floxed exon 3 activating allele of β-catenin (*Ctnnb1*^*tm1Mmt/+*^) [20] with mice expressing Cre recombinase under control of the pancreatic and duodenal homeobox 1 (*Pdx1*) promoter (*Pdx1-Cre* mice)[18]. Confirming previous studies [11], we found that newborn mutant *Pdx1-Cre; Ctnnb1*^*tm1Mmt/+*^ mice displayed a dramatic reduction of pancreatic tissue in both the splenic and gastric lobes (S1 Fig). The pancreatic remnant was clearly distinguishable from adjacent stomach and duodenum (S1 Fig). Histological and immunofluorescence analysis revealed the presence of epithelial cysts and abundant stroma in the pancreatic remnant (S1 Fig). Scarce pancreatic tissue with normal appearance could also be observed (S1 Fig). However, in these cells, β-catenin localization was restricted to the membrane with no apparent nuclear or cytoplasmic accumulation (S1 Fig) likely due to inefficient excision of the *Ctnnb1*^*tm1Mmt/+*^ transgene by the Cre recombinase.

**Fig 1:**
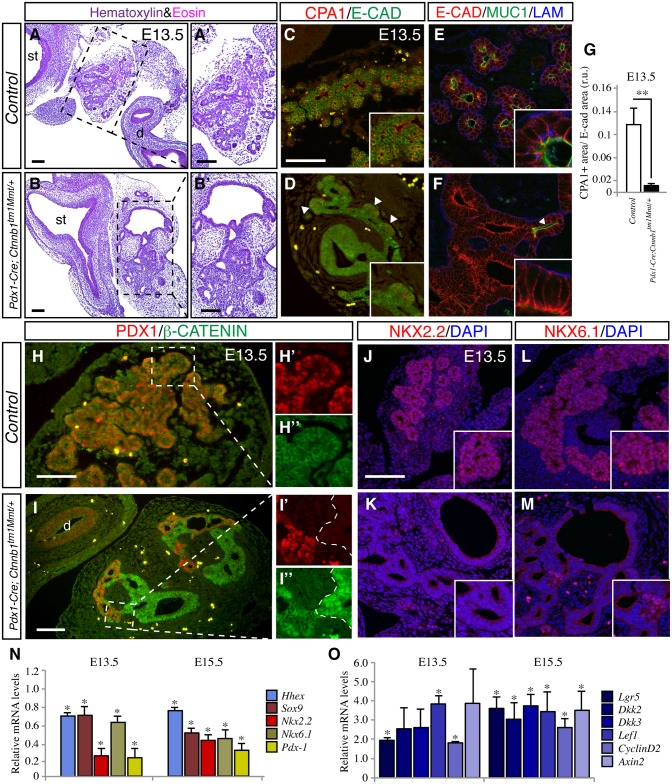
Loss of pancreatic cell identity in *Pdx1-Cre; Ctnnb1*^*tm1Mmt/+*^ pancreas. Hematoxylin/eosin staining of E13.5 pancreatic sections showing reduced branching and multiple dilations in the pancreatic epithelium of *Pdx1-Cre; Ctnnb1*^*tm1Mmt/+*^ pancreata (A) compared to control pancreata (B). Boxed areas in A and B are shown in higher magnification in A' and B', respectively. The enzyme Carboxipeptidase A1 (Cpa1, red) is located in the acinar progenitor cell population found at the tips of the E13.5 branching epithelium stained for the epithelial maker E-cadherin (green) in control mice (C). Cpa1-positive cells (arrowheads) are reduced in E13.5 *Pdx1-Cre; Ctnnb1*^*tm1Mmt/+*^ pancreatic epithelium (D). The pancreatic ductal epithelium in E13.5 *Pdx1-Cre; Ctnnb1*^*tm1Mmt/+*^ embryos display loss of apical marker Muc-1 (green) (arrowheads) but normal basal (laminin, blue) and basolateral marker (E-cadherin, green) in dilated areas (F) compared to control pancreata (E). Insets show higher magnification pictures. (G) Quantification of Cpa1-positive area. Marked reduction in Pdx-1 expression in *Pdx1-Cre Ctnnb1*^*tm1Mmt/+*^ pancreatic epithelium (I) at E13.5 compared to control pancreata (H). Note that in *Pdx1-Cre; Ctnnb1*^*tm1Mmt/+*^ pancreatic epithelium, Pdx-1 expression is dramatically reduced in cells displaying nuclear β-catenin accumulation while cells with only membranous β-catenin localization express significant Pdx-1 levels (I' and I''). Decreased expression of the embryonic pancreatic transcription factors NKX2.2 (J, K) and NKX6.1 (L, M) in E13.5 *Pdx1-Cre; Ctnnb1*^*tm1Mmt/+*^ pancreata. (N) Quantitative RT-PCR analysis of the expression of pancreatic transcription factors in E13.5 and E15.5 embryonic pancreas. (O) Quantitative RT-PCR analysis of the expression of canonical *Wnt* signaling target genes at E13.5 and E15.5 embryonic pancreas. Results in N and O are expressed as fold relative to levels in control pancreata (n = 3–6 embryos from each genotype). Data are presented as mean ± SEM; * p < 0.05 (Student's *t*-test). Scale bars 100 μm, (in A for A-B; in E for E-H; in I for I-J).

To determine the specific period in which β-catenin activation impaired embryonic pancreas formation, we analyzed *Pdx1-Cre; Ctnnb1*^*tm1Mmt/+*^ pancreata at various developmental stages. The first phenotypes were observed between E12.5-E13.5, with the pancreatic epithelium of *Pdx1-Cre; Ctnnb1*^*tm1Mmt/*^ embryos displaying abnormal lumen dilations (Fig 1A and 1B). Between E13.5 and E15.5, a dramatic morphogenetic remodeling occurs in the pancreatic epithelium that results in the formation of different progenitor domains, with proacinar progenitor cells located at the tips of the branching epithelium and ductal-endocrine precursors located along the epithelial trunk [26]. At E13.5, the control pancreas formed a branched epithelial (marked by E-Cadherin expression) tree with tips positive for Carboxypeptidase A1 (Cpa1) (Fig 1C). In contrast, the *Pdx1-Cre; Ctnnb1*^*tm1Mmt/+*^ pancreatic epithelium did not branch properly with a decrease in Cpa1-positive tips and abnormally expanded lumens (Fig 1D and 1G). The dilated epithelial areas were associated with loss of apical ductal markers such as Mucin-1 [27] (Fig 1E and 1F), although localization of basal and basolateral ductal markers laminin and E-cadherin was not affected (Fig 1E and 1F). By E15.5, the disorganization of the pancreatic epithelium and the lack of normal pancreatic tissue in *Pdx1-Cre; Ctnnb1*^*tm1Mmt/+*^ embryos were clearly apparent (S2 Fig). The epithelial dysmorphogenesis of *Pdx1-Cre; Ctnnb1*^*tm1Mmt/+*^ embryonic pancreata was associated with defective cell differentiation. Immunofluorescence and quantitative RT-PCR analyses of embryonic pancreata revealed a significant decrease in the expression of transcription factors essential for pancreas formation including Pdx1 (Fig 1H, 1I and 1N), Nkx2.*2* (Fig 1J, 1K and 1N), Nkx6.1 (Fig 1L, 1M and 1N), Ptf1a and Ngn3 (S2 Fig), Hhex and Sox9 (Fig 1N). Cells with normal levels of these pancreatic transcription factors were also found in *Pdx1-Cre; Ctnnb1*^*tm1Mmt/+*^ pancreata but, similar to what was observed in newborn pancreata, no nuclear β-catenin localization was observed (Fig 1I). To further characterize the molecular changes induced by β-catenin activation, we performed whole transcriptome analysis on *Pdx1-Cre; Ctnnb1*^*tm1Mmt/+*^ pancreata. Microarray analysis confirmed a marked downregulation of pancreatic genes in *Pdx1-Cre; Ctnnb1*^*tm1Mmt/+*^ E13.5 embryonic pancreata (Table 1). Consistent with the prominent nuclear accumulation of β-catenin, an increase in the expression of canonical Wnt signaling target genes was observed in *Pdx1-Cre; Ctnnb1*^*tm1Mmt/+*^ embryonic pancreata indicating hyperactivation of the canonical Wnt signaling pathway (Fig 1O). Thus, ectopic β-catenin activation in the developing mouse pancreas severely impairs pancreatic epithelial tubulogenesis and cell differentiation.

**Table 1:** Representative list of selected pancreatic genes downregulated in in *Pdx1-Cre; Ctnnb1*^*tm1Mmt/+*^ E13.5 embryonic pancreata.

Gene	Fold change	p-value
Neurog3	-24.49	1.68E-07
Pdx1	-17.83	2.67E-09
Neurod1	-15.87	1.81E-07
Cpa1	-14.51	2.74E-08
Slc2a2	-10.06	5.49E-06
Nr5a2	-9.65	2.27E-08
Nkx2-2	-8.93	1.16E-08
Muc1	-7.86	8.50E-07
Onecut1	-7.72	1.36E-07
Iapp	-6.64	8.52E-04
Bhlha15	-6.45	3.40E-04
Pax6	-6.11	7.48E-07
Nkx6-1	-5.72	1.59E-07
Rbpjl	-5.01	1.99E-04
Ptf1a	-4.93	2.68E-05
Hnf4a	-4.89	1.55E-06
Glis3	-3.60	7.70E-07
Rfx6	-3.28	2.33E-05
Gata4	-2.77	1.26E-04
Myt1	-2.71	4.40E-04
Hhex	-2.52	8.68E-05
Hnf1a	-2.49	3.69E-05

### Canonical Wnt signaling activation leads to activation of gastric and intestinal gene expression

**Fig 2:**
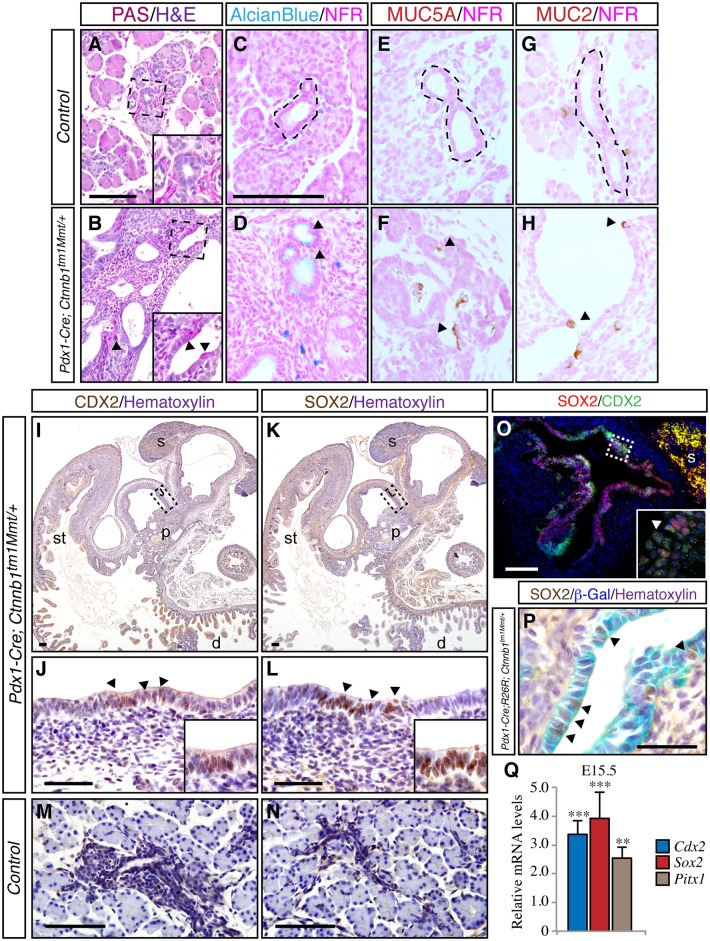
β-catenin stabilization induces activation of gastrointestinal genes. Staining for PAS (A, B) and Alcian Blue (C, D) in *Pdx1-Cre; Ctnnb1*^*tm1Mmt/+*^ and control newborn pancreata. Immunohistochemical staining for the gastric-specific mucin Muc5ac (E, F), intestine-specific mucin Muc2 (G, H) in control and mutant *Pdx1-Cre; Ctnnb1*^*tm1Mmt/+*^ newborn pancreata. Arrowheads mark cells lining the epithelium positive for the gastrointestinal markers. Cdx2 accumulation in pancreatic cysts of newborn *Pdx1-Cre; Ctnnb1*^*tm1Mmt/+*^ mice in low (I) and high (J) magnification pictures. Cdx2 is not detected in newborn control pancreata (M). Sox2 accumulation in pancreatic cysts of newborn *Pdx1-Cre; Ctnnb1*^*tm1Mmt/+*^ mice in low (K) and high (L) magnification pictures. No expression of Sox2 is detected in control pancreata (N). The boxed areas in I and K are shown at higher magnification in J and L, respectively. Arrowheads in J and L indicate the areas shown in the insets at higher magnification. (O) Coexpression of Sox2 and Cdx2 in epithelial cells of pancreatic cysts in newborn *Pdx1-Cre; Ctnnb1*^*tm1Mmt/+*^ mice. The boxed area in O is shown at higher magnification in the inset. (P) Coexpression of β-galactosidase and Sox2 gastric marker in epithelial cells (arrowheads) of pancreatic cysts in newborn *Pdx1-Cre; R26R; Ctnnb1*^*tm1Mmt/+*^ mice. (Q) Analysis of the expression level of transcription factors specific of intestine (Cdx2) and stomach (Sox2 and Pitx1) showed a significant increase in expression in the pancreas of E15.5 *Pdx1-Cre; Ctnnb1*^*tm1Mmt/+*^ embryos. Results are expressed as fold relative to levels in control pancreata (n = 6 embryos from each genotype). Data are presented as mean ± SEM; ** p < 0.01 *** p < 0.001 (Student's *t*-test). st, stomach; d, duodenum. Scale bars, 100 μm.

The cystic structures present in newborn *Pdx1-Cre; Ctnnb1*^*tm1Mmt/+*^ pancreata were lined by a single-layered cuboidal epithelium surrounded by dense mesenchyme (S1 Fig), features that are reminiscent of gut-like structures. To define the nature of the cystic structures, we examined a number of gastric and intestinal markers in newborn *Pdx1-Cre; Ctnnb1*^*tm1Mmt/+*^ pancreata. Alcian blue and periodic acid Schiff (PAS) staining revealed the presence of cells abnormally producing mucin in the pancreatic epithelium (Fig 2A–2D). In agreement with these observations, cells expressing gastric-specific mucin Muc5ac (Fig 2E and 2F) and cells expressing intestine-specific mucin Muc2 (Fig 2G and 2H) were found interspersed within the pancreatic epithelium of *Pdx1-Cre; Ctnnb1*^*tm1Mmt/+*^ mice. To further characterize the cystic structures of *Pdx1-Cre Ctnnb1*^*tm1Mmt/+*^ pancreata, we studied embryonic gastric and intestinal markers. Sox2 and Cdx2 are transcription factors essential for the formation of stomach and intestine, respectively [28, 29]. During embryonic development, Sox2 and Cdx2 are exclusively expressed in the foregut and intestinal lineages, respectively, thus permitting a clear distinction between stomach and intestine [30]. In newborn control pancreata, neither Sox2 nor Cdx2 was expressed in the pancreatic epithelium as assessed by immunohistochemical analysis (Fig 2M and 2N). However, a strong Sox2 signal was observed in the epithelial compartment of cysts in *Pdx1-Cre; Ctnnb1*^*tm1Mmt/+*^ pancreata (Fig 2K and 2L). Interestingly, cells positive for the intestinal transcription factor Cdx2 were also found within the pancreatic epithelial cysts of *Pdx1-Cre; Ctnnb1*^*tm1Mmt/+*^ mice (Fig 2I and 2J). Colocalization experiments with Sox2 and Cdx2 revealed that cells of *Pdx1-Cre; Ctnnb1*^*tm1Mmt/+*^ pancreata coexpress both markers (Fig 2O). To confirm the origin of the epithelial cysts of *Pdx1-Cre; Ctnnb1*^*tm1Mmt/+*^ pancreata, we performed genetic lineage tracing experiments using *Pdx1-Cre* to irreversibly activate the expression of the reporter *R26R* (ß-galactosidase). X-gal staining of newborn *Pdx1-Cre; R26R; Ctnnb1*^*tm1Mmt/+*^ pancreata revealed that the epithelial cells lining the cysts were positive for β-galactosidase (S1 Fig), a result consistent with a pancreatic origin. Immunohistochemistry analysis on X-gal-stained sections revealed that the Pdx1-derived cells expressed gut markers such as Sox2 (Fig 2P). Consistent with the immunohistochemical data, microarray analysis revealed a prominent increase in stomach progenitor genes such as Sox2, Pitx1 and Barx1 (Table 2 and Fig 2Q) and intestinal progenitor genes such as Cdx2, Lgr5 and FoxL (Table 2 and Fig 2Q) indicating aberrant activation of the gastrointestinal genetic program during early embryonic stages. Summarily, these results show that β-catenin activation in the pancreatic primordium results in loss of pancreatic identity with pancreatic cells adopting the morphological and molecular features of gut-like cells.

**Table 2:** Representative list of selected intestinal and gastric genes upregulated in in *Pdx1-Cre; Ctnnb1*^*tm1Mmt/+*^ E13.5 embryonic pancreata.

Gene	Fold change	p-value	Normal expression domain
Lgr5	21.75	3.54E-09	Intestinal stem cells
Foxf2	16.06	4.03E-07	Gut mesenchyme
Foxf1a	11.63	1.18E-08	Gut mesenchyme
Gata5	3.35	3.96E-06	Heart and gut
FoxL1	3.31	3.47E-06	Gut mesenchyme
Myocd	3.26	2.25E-06	Gut mesenchyme
Acta2	2.83	1.67E-06	Smooth muscle
Nkx2-3	2.12	1.72E-04	Gastrointestinal mesenchyme
Pitx1	1.99	5.21E-04	Stomach
Cdx2	1.97	3.32E-05	Intestine
Sox2	1.61	3.29E-03	Stomach
Barx1	2.39	7.74E-05	Stomach mesenchyme

### Elevated canonical Wnt signaling causes a dramatic reorganization of the surrounding mesenchyme

**Fig 3:**
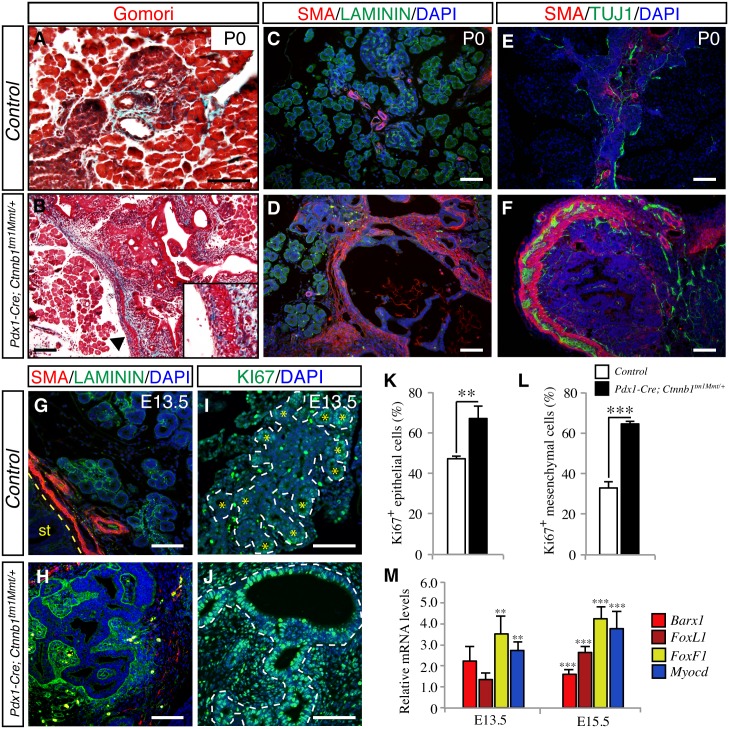
Increased mesenchyme in pancreas with activated β-catenin signaling. Gomori staining shows increased mesenchyme surrounding the epithelium of the pancreatic cystic structures in newborn *Pdx1-Cre; Ctnnb1*^*tm1Mmt/+*^ mice (B) compared to control mice (A). Arrowheads in B indicate the region enlarged in the inset. Immunofluorescence for antibodies against laminin and smooth muscle actin (SMA) suggest formation of smooth muscle in newborn *Pdx1-Cre; Ctnnb1*^*tm1Mmt/+*^ pancreas (D). SMA-positive layers are only found associated with big blood vessels in control pancreata (C). Tuj1-positive cells are intercalated in the SMA-positive layer surrounding the cysts in newborn *Pdx1-Cre Ctnnb1*^*tm1Mmt/+*^ pancreata (F) compared to control pancreata (E). Increased pancreatic mesenchyme in *Pdx1-Cre Ctnnb1*^*tm1Mmt/+*^ embryos (H) compared to control embryos (G) is observed during early stages of pancreas formation. Increased proliferation (marked by Ki-67 immunostaining) of epithelial and mesenchymal cells in E13.5 *Pdx1-Cre; Ctnnb1*^*tm1Mmt/+*^ embryonic pancreata (J) compared to control pancreata (I). Note that proliferative epithelial cells in control embryonic pancreata are preferentially located at the tips of the branching epithelium (yellow asterisks). Pancreatic epithelium is outlined in white dashed line. Quantification of proliferating epithelial (K) and mesenchymal (L) cells in E13.5 embryonic pancreas. Results are shown as percentage of KI67^+^ cells. (n = 3 embryos from each genotype). (M) Analysis of the expression level of mesenchymal gastrointestinal markers shows a significant increase in expression in the pancreas of E13.5 and E15.5 *Pdx1-Cre; Ctnnb1*^*tm1Mmt/+*^ embryos. Results are expressed as fold relative to levels in control pancreata (n = 3–6 embryos from each genotype). Data in K, L and M are presented as mean ± SEM; ** p< 0.01, *** p< 0.001 (Student's *t*-test). Scale bars 100 μm.

One of the most distinctive features of *Pdx1-Cre; Ctnnb1*^*tm1Mmt/+*^ pancreata in newborn mice was the dramatic increase in mesenchymal tissue (S1 Fig). Trichrome staining revealed a thick mesenchyme layer surrounding the epithelium of the cystic structures in newborn *Pdx1-Cre; Ctnnb1*^*tm1Mmt/+*^ mice in contrast to the scarce mesenchymal tissue present in control mice (Fig 3A and 3B). To further characterize this expanded mesenchymal tissue, immunofluorescence analysis was performed with antibodies against laminin and smooth muscle actin (SMA). In control pancreas, SMA was found only in big blood vessels while laminin was detected surrounding all the epithelial structures (Fig 3C and 3E). Interestingly, in *Pdx1-Cre; Ctnnb1*^*tm1Mmt/+*^ pancreata, the cystic structures were surrounded by a basement membrane and a thick layer of mesenchyme tissue positive for SMA (Fig 3D and 3F). A bilayer of SMA-positive cells with intercalated Tuj1-positive cells (indicative of the presence of neural cells), features characteristic of gut mesenchyme, was observed in some big pancreatic cysts in *Pdx1-Cre; Ctnnb1*^*tm1Mmt/+*^ mice (Fig 3F). This dramatic expansion of condensed mesenchyme seemed to be due to non-cell autonomous effects, secondary to β-catenin activation in the epithelial compartment, as suggested by the lack of nuclear β-catenin in mesenchymal cells of *Pdx1-Cre; Ctnnb1*^*tm1Mmt/+*^ pancreata (S1 Fig). Indeed, X-gal staining of *Pdx1-Cre; R26R; Ctnnb1*^*tm1Mmt/+*^ pancreata revealed that cells positive for β-galactosidase were found only in the epithelial compartment (S1 Fig), ruling out an epithelial-mesenchymal transition phenomenon. Analysis of embryonic stages revealed expansion of SMA-positive mesenchymal cells as early as E13.5, concordant with the formation of dilated tubular epithelial structures (Fig 3G and 3H). At this stage, a significant increase in the proliferation of both mesenchymal and epithelial cells of embryonic *Pdx1-Cre; Ctnnb1*^*tm1Mmt/+*^ pancreata was noted (Fig 3I–3L). Consistent with the morphological and immunohistochemical data indicating expansion of gut-like mesenchyme in embryonic *Pdx1-Cre; Ctnnb1*^*tm1Mmt/+*^ pancreata, microarray analysis and quantitative PCR analysis revealed increased expression of several transcriptional regulators linked to gastrointestinal mesenchyme differentiation including Nkx2-3, FoxF1, FoxL1, Barx1 and myocardin (specific to the smooth muscle cell lineage)[31, 32] (Table 2 and Fig 3M). Of note, Barx1 is a transcription factor specifically expressed in stomach mesenchyme during embryonic development, consistent with the increased Sox2 and Pitx1 expression observed in *Pdx1-Cre; Ctnnb1*^*tm1Mmt/+*^ embryonic pancreata. Thus, activation of the canonical Wnt pathway in the pancreatic epithelial compartment results in the acquisition by surrounding mesenchyme of features usually associated with gastrointestinal lineages, illustrating a dramatic regulatory role of the epithelium on the mesenchyme during embryonic development.

### ß-catenin activation in the foregut endoderm severely impairs pancreas formation

**Fig 4:**
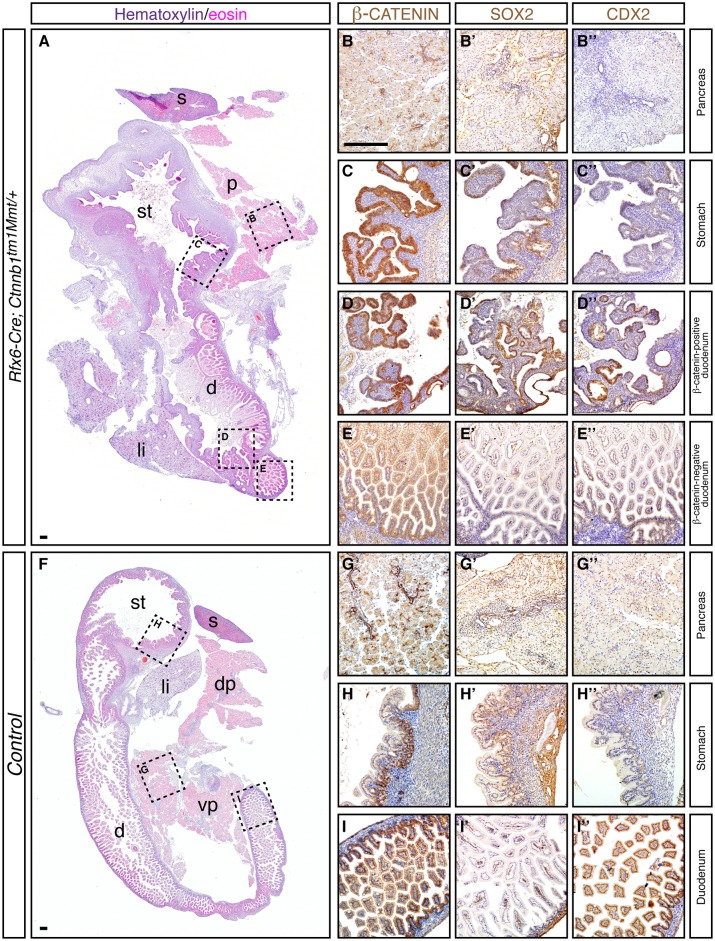
Early β-catenin activation in foregut endoderm severely impacts pancreas formation. Low magnification pictures of hematoxylin/eosin staining of paraffin sections of E18.5 *Rfx6-Cre; Ctnnb1*^*tm1Mmt/+*^ (A) and control mice (F). Boxed areas are regions that are shown in higher magnification in right panels for each antibody marker used in consecutive paraffin sections. The pancreatic remnant of E18.5 *Rfx6-Cre; Ctnnb1*^*tm1Mmt/+*^ mice displayed membranous β-catenin localization (B) and no accumulation of Sox2 (B') and Cdx2 (B''). The stomach of E18.5 *Rfx6-Cre; Ctnnb1*^*tm1Mmt/+*^ mice displayed nuclear β-catenin localization (C), expression of Sox2 (C') but not Cdx2 (C'). The dilated area of the duodenum located proximal to the stomach (D) displayed nuclear β-catenin accumulation concomitant with increased expression of Sox2 (D') and Cdx2 (D''). However, the duodenal area distal to the stomach did not display nuclear β-catenin localization (E). In this area, Cdx2 (E'') but not Sox2 (E'), was expressed. In E18.5 control mice, pancreas (G), stomach (H) and duodenum (I) displayed β-catenin membranous localization. No Sox2 (G') or Cdx2 (G'') was expressed in control pancreas. In control stomach, Sox2 was expressed (H') but not Cdx2 (H''). In control duodenum, Cdx2 was expressed (I'') but not Sox2 (I'). d, duodenum; dp, dorsal pancreas; li, liver; p, pancreas, spleen; st, stomach; vp, ventral pancreas. Scale bars 100 μm (in B for B-I).

Our results indicate that β-catenin activation in the Pdx-1 domain redirects the developmental fate of the embryonic pancreas towards a gastrointestinal phenotype, however, the formation of the pancreatic bud is initiated in *Pdx1-Cre; Ctnnb1*^*tm1Mmt/+*^ mice (Fig 1A–1D). Since canonical Wnt signaling has been implicated in patterning of the primitive gut endoderm [16, 33, 34], we wanted to determine whether β-catenin activation could affect the initiation of pancreatic bud outgrowth in the foregut endoderm. To this end, we crossed *Ctnnb1*^*tm1Mmt/+*^ mice with mice expressing Cre recombinase under control of the *Rfx6* promoter [19]. *Rfx6-Cre* is broadly expressed in the early gut endoderm, thus allowing the activation of β-catenin prior to pancreatic bud initiation. Gross morphological examination of *Rfx6-Cre; Ctnnb1*^*tm1Mmt/+*^ embryos at late gestation (E18.5) revealed a hypoplastic pancreas lacking the normal gross morphology (S3 Fig). The splenic lobe appeared greatly reduced in size and the gastric lobe was almost completely absent. Indeed, the main pancreatic duct could not be identified in the pancreatic remnant of *Rfx6-Cre; Ctnnb1*^*tm1Mmt/+*^ embryos. The stomach and spleen appeared morphologically unaltered but the rostral-most duodenum was greatly dilated (S1 Fig). Histological analysis of the hypoplastic pancreata did not reveal major morphological changes compared with control tissues (Fig 4A and S3 Fig). Immunofluorescent analysis for the different epithelial cell lineages (acinar, ductal, and islet) within the pancreas revealed normal exocrine tissue (S3 Fig) but a scarcity of islet structures was noted (S3 Fig). β-catenin immunohistochemistry revealed that the remnant pancreatic tissue of *Rfx6-Cre; Ctnnb1*^*tm1Mmt/+*^ embryos was entirely comprised of cells in which β-catenin localization was restricted to the membrane with no nuclear or cytoplasmic localization (Fig 4B and S5 Fig). Thus, the pancreatic remnant in *Rfx6-Cre; Ctnnb1*^*tm1Mmt/+*^ embryos seems to arise from cells that fail to undergo Cre-mediated recombination indicating that pancreatic cells with active β-catenin do not contribute to the developing pancreas. The analysis of pancreas of *Rfx6-Cre; Ctnnb1*^*tm1Mmt/+*^ embryos at early stages of development corroborated this notion. A dramatic reduction in pancreatic epithelium, as assessed by immunohistochemistry for Cpa1 and Pdx-1 pancreatic markers, was observed in *Rfx6-Cre; Ctnnb1*^*tm1Mmt/+*^ pancreatic buds (S3 and S4 Figs). Furthermore, no nuclear β-catenin localization was detected in this remnant pancreatic epithelium indicating that β-catenin activation blocks pancreatic cell formation at the early stages of pancreas development.

The rostral-most duodenum of *Rfx6-Cre; Ctnnb1*^*tm1Mmt/+*^ embryos appeared dilated and morphologically different from the posterior duodenum (Fig 4A). Cross-sections of the dilated duodenum revealed morphologically normal villi in the region distal to the stomach. However, the area proximal to the stomach was markedly different with disorganized villi (Fig 4A). Interestingly, clear nuclear β-catenin accumulation was observed in the epithelial cells of the dilated duodenal structure, but only in those proximal to the stomach (Fig 4D and S5 Fig). The cells located in the otherwise morphologically normal duodenum exhibited membranous β-catenin localization (Fig 4E and S5 Fig). The dilated duodenal structures of *Rfx6-Cre; Ctnnb1*^*tm1Mmt/+*^ embryos are morphologically reminiscent of the cystic structures present in newborn *Pdx1-Cre; Ctnnb1*^*tm1Mmt/+*^ pancreata. To define the nature of the dilated rostral-most duodenum of *Rfx6-Cre; Ctnnb1*^*tm1Mmt/+*^ embryos, we studied embryonic gastric and intestinal markers. We performed immunohistochemistry for Sox2 and Cdx2 markers in the dilated rostral-most duodenum of *Rfx6-Cre; Ctnnb1*^*tm1Mmt/+*^ embryos Surprisingly, we observed mixed accumulation of Sox2 and Cdx2 in the region that showed prominent nuclear β-catenin (Fig 4D' and 4D'') and thus, losing the clear boundary between stomach and intestine. No Sox2 or Cdx2 accumulation was observed in either the remnant pancreatic epithelium of *Rfx6-Cre; Ctnnb1*^*tm1Mmt/+*^ embryos (Fig 4B' and 4B'') or control pancreata (Fig 4G' and 4G''). These histological and immunohistochemistry observations suggest that the region in *Rfx6-Cre; Ctnnb1*^*tm1Mmt/+*^ embryos destined to form the pancreatic foregut was located in the stomach-duodenum boundary and developed a mixed gastric-and-intestinal identity, although we cannot formally prove it without proper lineage tracing methods (non-Cre dependent).

### Inhibition of the Hedgehog pathway partially rescues organogenesis in pancreas with hyperactivation of canonical Wnt signaling

**Fig 5:**
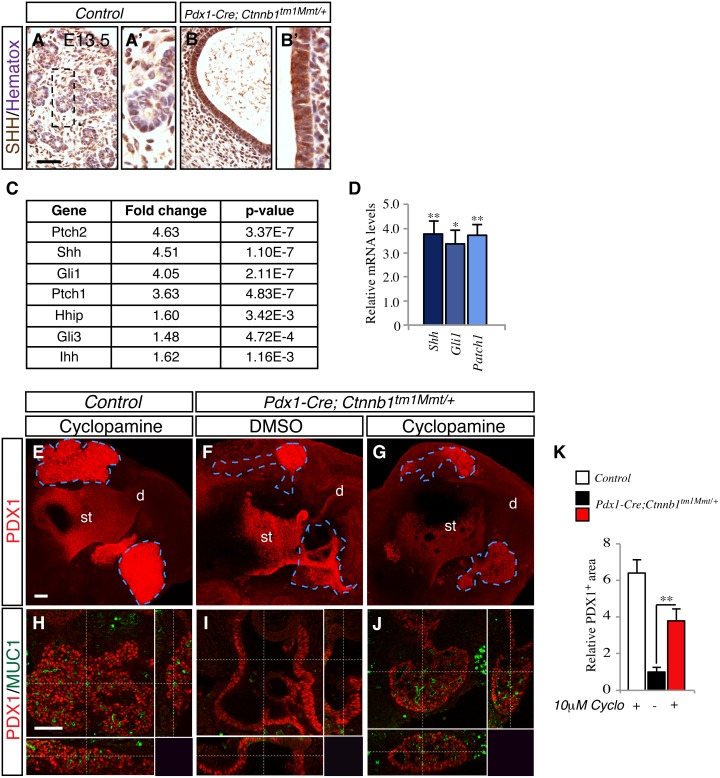
Inhibition of the Hedgehog pathway partially rescues pancreas formation in pancreas with activated β-catenin signaling. Increased Shh expression in the embryonic (E13.5) epithelium of *Pdx1-Cre; Ctnnb1*^*tm1Mmt/+*^ pancreas (B) compared to control pancreata (A). The boxed areas are shown at higher magnification in the adjacent panels (A' and B'). (C) Fold change and p-values from upregulated components of the hedgehog pathway in E13.5 *Pdx1-Cre; Ctnnb1*^*tm1Mmt/+*^ pancreas observed in microarray analysis. (D) Increase in expression of Hh pathway genes in embryonic E15.5 *Pdx1-Cre; Ctnnb1*^*tm1Mmt/+*^ pancreata as assessed by quantitative PCR. Results are expressed as fold relative to levels in control pancreata (n = 6 embryos from each genotype). Data are presented as mean ± SEM; *p < 0.05 **p < 0.01 (Student's *t*-test). (E-J) Hh inhibition partially rescues pancreas formation in cultured pancreatic rudiments. Images show confocal *z*-stacks of pancreatic explants in whole-mounts stained for Pdx1. Foreguts from E10.5 *Pdx1-Cre; Ctnnb1*^*tm1Mmt/+*^ and control mice were cultured in the presence or absence of 10 μM cyclopamine for 3 consecutive days. Vehicle-treated *Pdx1-Cre Ctnnb1*^*tm1Mmt/+*^ pancreatic buds displayed a reduced and disorganized epithelium positive for Pdx-1 (F) compared to control pancreatic buds (E). (G) Cyclopamine-treated *Pdx1-Cre; Ctnnb1*^*tm1Mmt/+*^ pancreatic buds displayed an increase in Pdx-1-positive epithelium and a recovery of the normal epithelium morphology. (H-J) Whole-mount immunostaining for Muc-1 (green) and Pdx-1 (red) showing reduced branching and decreased Muc-1 expression in vehicle-treated *Pdx1-Cre; Ctnnb1*^*tm1Mmt/+*^ pancreatic buds (I) compared to control cyclopamine-treated control pancreatic buds (H). Addition of cyclopamine partially rescued these phenotypes (J). Images are *z*-stacks of serial confocal sections. (K) Quantification of Pdx-1-positive pancreatic epithelium in cultured explants. Results are shown as normalized ratios compared to DMSO-treated mutant *Pdx1-Cre; Ctnnb1*^*tm1Mmt/+*^ pancreatic explants (*n* = 6 explants per group, 3 independent experiments). Data are presented as mean ± SEM. ** p< 0.01 (one-way ANOVA). Scale bars 100 μm, (in A for A-B; in E for E-G; in H for H-J).

The Wnt and Hedgehog (Hh) signaling pathways interact at multiple levels during the development of the gastrointestinal tract [33, 35, 36]. Increased upregulation of Hh ligands has been previously described in *Pdx1-Cre; Ctnnb1*^*tm1Mmt/+*^ pancreata [11]. Interestingly, overexpression of the Hh ligand *Shh* in the Pdx1-expressing domain results in the formation of epithelial cysts and pancreatic to intestinal transformation [37], a phenotype similar to what we have observed in pancreata with β-catenin overactivation. We decided to test whether Hh signaling mediated the pancreatic phenotypes due to β-catenin overactivation by inhibiting the Hh pathway by pharmacological means. First, and confirming previous studies [11], we found increased *Shh* expression in embryonic *Pdx1-Cre; Ctnnb1*^*tm1Mmt/+*^ pancreata by immunohistochemistry (Fig 5A and 5B) and quantitative PCR (Fig 5D). Furthermore, increased expression of the Shh target genes, *Ptch1* and *Gli1*, was also found indicating increased Hh activity (Fig 5D). To more directly test the hypothesis that Hh signaling mediates the developmental defects in pancreas with activated β-catenin, we studied embryonic pancreatic rudiments cultured with cyclopamine, an inhibitor of Hh signaling that binds to Smoothened [38]. E10.5 foreguts from *Pdx1-Cre; Ctnnb1*^*tm1Mmt/+*^ and control mice were dissected and cultured with cyclopamine (10μM) or vehicle (DMSO) for 3 consecutive days. Whole-mount immunohistochemistry for Pdx1 was performed in the pancreatic explants to measure pancreatic epithelial area. In agreement with the immunohistochemistry data obtained in paraffin sections of embryonic *Pdx1-Cre; Ctnnb1*^*tm1Mmt/+*^ pancreata (Fig 1I), a marked reduction in Pdx1-stained area was observed in vehicle-treated *Pdx1-Cre; Ctnnb1*^*tm1Mmt/+*^ pancreatic explants compared with cyclopamine-treated control pancreatic explants (Fig 5E, 5F, 5H, 5I and 5K). Addition of cyclopamine to *Pdx1-Cre; Ctnnb1*^*tm1Mmt/+*^ pancreatic rudiments partially rescued this phenotype, significantly increasing the pancreatic epithelial area (Fig 5G, 5J and 5K). To confirm this observation, we performed whole-mount immunohistochemistry for Pdx1 and Mucin-1, a marker that allows sharp visualization of the embryonic pancreatic epithelium. As expected from the above data, a disorganized epithelium with dilations was observed in vehicle-treated *Pdx1-Cre Ctnnb1*^*tm1Mmt/+*^ pancreas explants compared to the densely packed epithelium of vehicle-treated control pancreatic explants (Fig 5H and 5I). Furthermore, the dilated epithelial areas displayed loss of Mucin-1 expression (Fig 5I) as we had observed in pancreata of *Pdx1-Cre; Ctnnb1*^*tm1Mmt/+*^ embryos (Fig 1H). Cyclopamine treatment of *Pdx1-Cre; Ctnnb1*^*tm1Mmt/+*^ pancreatic rudiments partially restored these phenotypes. No major dilations were observed in the pancreatic epithelium and the accumulation pattern of Muc-1 was similar to that found in control explants (Fig 5J). Taken together, these results support a key role of Hh signaling in the developmental defects of pancreas due to β-catenin activation.

## Discussion

Previous studies have shown that activation of canonical Wnt signaling in the embryonic stages of pancreas formation results in severe pancreatic hypoplasia [11, 13, 15]. Here, we show that activation of β-catenin in pancreatic progenitors causes aberrant induction of gastric and intestinal markers in the pancreatic epithelium and mesenchyme, leading to the development of gut-like features. This β-catenin-induced block of pancreas formation depends, at least partly, on Hh signaling.

Our results further extend previous studies showing that repression of the canonical branch of the Wnt signaling pathway in the very early stages of pancreas development is essential for proper pancreas formation in mice [11]. In Xenopus embryos, induction of Wnt/β-catenin signaling in anterior endoderm blocks pancreas development [39]. In agreement with these studies, activation of β-catenin in the prepancreatic endoderm using the *Rfx6-Cre* line impairs pancreas formation. The pancreatic epithelium is markedly reduced at early stages of pancreas development and at birth in *Rfx6-Cre; Ctnnb1*^*tm1Mmt/+*^ mice. Furthermore, no nuclear or cytoplasmic accumulation of β-catenin is detected in the scarce normal pancreatic tissue indicating that it is composed of cells that have arisen from progenitors that escaped Cre-mediated excision of the *Ctnnb1*^*tm1Mmt/+*^ allele.

Activation of β-catenin has also a profound impact on embryonic pancreas development after the pancreatic bud growth has initiated. In *Pdx1-Cre; Ctnnb1*^*tm1Mmt/+*^ embryos, activation of β-catenin occurs after endodermal progenitors have acquired a pancreatic fate. Starting at E12.5, we found that *Pdx1-Cre; Ctnnb1*^*tm1Mmt/+*^ pancreata exhibit a dramatic reduction in the expression of transcription factors known to be required for pancreas formation. Also, epithelial branching morphogenesis and differentiation of the exocrine and endocrine lineages is markedly compromised. Concomitant with the downregulation of key pancreatic transcription factors, ectopic expression of gastric and intestinal genes is observed. At birth, pancreata of *Pdx1-Cre; Ctnnb1*^*tm1Mmt/+*^ mice were comprised of cystic structures with features typical of stomach/intestine, including cuboidal epithelium, accumulation of mucins and a layer of SMA in the underlying mesenchyme.

Wnt signaling plays a key role in intestinal specification and patterning [16]. It has been suggested that intestinal differentiation is the default program for the developing gut endoderm and that inhibition of canonical Wnt signaling is necessary for the formation of other foregut-derived organs [33]. Forced activation of canonical Wnt signaling in developing endoderm [16, 39], lung [40] and stomach [41] induces intestinal differentiation. Our results demonstrate that activation β-catenin in pancreatic progenitors also induces ectopic expression of intestinal genes such as the transcription factor Cdx2 or the intestine-specific mucin Muc2. Cdx2 is a transcription factor whose expression is very specific to the intestine and that plays a critical role in intestinal development [28, 30]. Expression of Cdx2 in foregut endoderm induces intestinal metaplasia [42]. Interestingly, induction of intestinal fate by ectopic Wnt signaling in endoderm seems to be mediated by Cdx2 [16]. In concordance with the pivotal role of Cdx2 in the induction of an intestinal genetic program, increased expression of Cdx2 was observed as early as E13.5 in *Pdx1-Cre; Ctnnb1*^*tm1Mmt/+*^ pancreata.

In addition to intestinal genes, activation of β-catenin resulted in the induction of gastric markers in the pancreatic domain. In particular, we observed ectopic expression of transcription factors that are essential for stomach development such as Sox2, Pitx1 and Barx1. Previous studies have shown that these markers are specific for the anterior part of the primitive gut [29, 31]. Barx1 is particularly interesting since its expression is restricted to the mesenchyme and exerts a non-cell autonomous effect on the overlying gut endoderm to regulate stomach development [41]. The induction of stomach genes upon β-catenin activation in embryonic pancreata seems to be in apparent contradiction with the above hypothesis that Wnt/β-catenin signaling promotes intestinal fate. One possible explanation to reconcile these observations comes from the proposed model for canonical Wnt signaling function in stomach development [33, 43]. Wnt signaling is active in the prospective stomach endoderm early in development, between E9.5 and E14.5 [43]. However, canonical Wnt signaling activity needs to be attenuated later to allow stomach differentiation. The inhibition of canonical Wnt signaling in stomach is achieved by the transcription factor Barx1 whose expression is restricted to the stomach mesenchyme and that induces the secretion of Wnt antagonists such as SFRPs [41]. Thus, although attenuation of canonical Wnt signaling is necessary for late stomach differentiation, a transient burst of canonical Wnt signaling activity might be necessary for early embryonic stomach development. We propose that activation of β-catenin in the pancreatic epithelium induces a gastric genetic program including expression of the mesenchymal transcription factor Barx1. Even though increased expression of Barx1 might induce secretion of Wnt antagonists, forced, irreversible β-catenin activation in the epithelium of *Pdx1-Cre; Ctnnb1*^*tm1Mmt/+*^ pancreata prevents a full transformation of the pancreas into stomach and consequently, inducing expression of intestinal markers. In concordance with this notion, the mixed gastric/intestinal phenotype observed in pancreata with activated β-catenin is reminiscent of the stomach phenotypes of Barx1-deficient mice as well as stomach of mice with activation of β-catenin in the prospective stomach endoderm [41].

The mix of gastric and intestinal markers induced by β-catenin activation in pancreatic cells together with the delay in the full activation of these markers (strong accumulation as assessed by immunohistochemistry is observed only during late embryonic stages of pancreas formation) raises the question of whether this can be considered a true fate conversion event. An alternative possibility is that activation of β-catenin blocks pancreas differentiation leaving the pancreatic cells in the default program for the developing gut endoderm resulting in the formation of aberrant gut progenitor cells that express both gastric and intestinal markers. In agreement with this notion, colocalization experiments revealed the presence of cells that simultaneously express Cdx2 and Sox2 in *Pdx1-Cre; Ctnnb1*^*tm1Mmt/+*^ pancreata.

Hh signaling is active and necessary for the embryonic development of the gut but excluded from the pancreas [35, 44]. Our experiments performed in vitro with pancreatic rudiment cultures demonstrate that Hh signaling at least in part mediates the pancreatic phenotypes due to β-catenin activation. Inhibition of the Hh pathway by the Smoothened inhibitor cyclopamine partially rescues pancreatic formation in *Pdx1-Cre; Ctnnb1*^*tm1Mmt/+*^ pancreatic rudiments. To this regard, the Hedgehog pathway plays an important role in the development of the gastrointestinal mucosa, including proliferation of mesenchymal progenitors and differentiation of the smooth muscle lineage [36], in agreement with the increased proliferation we observed in the mesenchymal compartment of pancreas with activated β-catenin.

The upregulation of the Hh pathway in embryonic pancreas induced by β-catenin activation is in concordance with previous studies describing the effects of overactivating the Hh pathway in the pancreas [37]. Thus, misexpression of the Hh ligand *Shh* in the pancreatic domain causes a pancreatic to intestinal transformation reminiscent of pancreata with activated β-catenin, including increased SMA-positive mesenchyme and epithelial cysts. Also, in vitro studies performed in pancreatic rudiments have shown that activin A causes differentiation of intestinal tissue from embryonic pancreas via a Hh-dependent mechanism [23]. Thus, our study provides a mechanistic framework for our and previously reported data regarding the role of these signaling pathways in gut development.

Our results provide additional evidence for the remarkable developmental plasticity of pancreatic progenitors [4]. Other studies have shown cell fate switches of the pancreatic epithelium, including pancreatic to intestinal [37, 45–47] and pancreatic to liver transformation [48]. However, this plasticity seems to be limited to the early stages of pancreas development. Activation of β-catenin signaling after E12.5 does not impair pancreas formation [11]. Although expression of gastric and intestinal markers was not specifically evaluated in these studies, the lack of morphological abnormalities in those pancreata indicate that the pancreatic epithelium is refractory to the effect of β-catenin in activation of the gastrointestinal genetic program. Interestingly, a similar time window for plasticity of the pancreatic epithelium has been reported for pancreas-to-liver transformation induced by loss of the transcription factor Sox9 [48]. This period coincides with the stage at which the pancreatic epithelium contains multipotent progenitor cells with the potential to give rise to all pancreatic cell types suggesting that this undifferentiated state is particularly sensitive to developmental plasticity.

Our study illustrates the importance of precise spatial and temporal control of the activity of embryonic signaling pathways for proper organ formation. Our work could provide insight into various human developmental anomalies of the pancreas such as pancreatic agenesis and pancreatic cysts [49]. Several mutations in genes encoding transcription factors have been associated with human developmental disorders of the human pancreas (reviewed in [1, 9]). However, little is known about the possible involvement of embryonic signaling pathways in these developmental anomalies. To this regard, cases of atrophy of the pancreas associated with cysts that are reminiscent of the cysts found in *Pdx1-Cre; Ctnnb1*^*tm1Mmt/+*^ mice have been reported [50, 51]. It would be interesting to analyze whether activation of Wnt/β-catenin signaling is observed in these malformations of the pancreas.

## Supporting Information

S1 Fig Pancreatic abnormalities in *Pdx1-Cre; Ctnnb1*^*tm1Mmt/+*^ newborn mice. (A, D) Whole mount pictures of representative P0 pancreata from *Pdx1-Cre; Ctnnb1*^*tm1Mmt/+*^ and control embryos. Low magnification pictures of Hematoxylin/eosin-stained paraffin sections of control (B) and *Pdx1-Cre; Ctnnb1*^*tm1Mmt/+*^ P0 mice (E). The boxed areas in B and E are shown at higher magnification in C, F and G. High magnification picture of control pancreas (C). High magnification picture of the scarce normal pancreatic tissue (F) and pancreatic cysts (G) in *Pdx1-Cre; Ctnnb1*^*tm1Mmt/+*^ mice. Immunohistochemistry reveals membranous localization of β-catenin in control pancreatic tissue (C') and in normal pancreatic tissue of *Pdx1-Cre; Ctnnb1*^*tm1Mmt/+*^ mice (F'). Nuclear β-catenin localization is found in epithelial cells of pancreatic cystic structures of *Pdx1-Cre; Ctnnb1*^*tm1Mmt/+*^ P0 mice (G'). Reduced islet formation *Pdx1-Cre; Ctnnb1*^*tm1Mmt/+*^ newborn mice (H) compared to control mice (I). Higher magnification pictures are shown in insets. (J) Genetic labeling of pancreatic cells by detection of reporter β-galactosidase activity in newborn P0 *Pdx1-Cre; R26R; Ctnnb1*^*tm1Mmt/+*^ pancreata. β-galactosidase was restricted to the epithelial compartment. Note that the epithelial cells lining the cysts were positive for β-galactosidase. Scale bars, 100 μm. d, duodenum; dp, dorsal pancreas; Li, liver st, stomach; vp, ventral pancreas.
(TIF)

S2 Fig Impairment of pancreatic differentiation in *Pdx1-Cre; Ctnnb1*^*tm1Mmt/+*^ embryos. Hematoxylin/eosin-stained paraffin sections of E15.5 control (A) and *Pdx1-Cre; Ctnnb1*^*tm1Mmt/+*^ pancreata (B). (D) Reduced PTF1a expression in E13.5 *Pdx1-Cre; Ctnnb1*^*tm1Mmt/+*^ embryonic pancreata compared to control embryos (C). (F) Reduced NGN3 expression in E13.5 *Pdx1-Cre; Ctnnb1*^*tm1Mmt/+*^ embryonic pancreata compared to control embryos (E). Higher magnification pictures are shown in insets. d, duodenum; dp, dorsal pancreas; p, pancreas; s, spleen; st, stomach; vp, ventral pancreas.
(TIF)

S3 Fig Histological analysis of embryonic *Rfx6-Cre; Ctnnb1*^*tm1Mmt/+*^ pancreata. Whole mount pictures of representative P0 pancreata from *Rfx-Cre; Ctnnb1*^*tm1Mmt/+*^ (A) and control mice (B). Pancreatic tissue is outline in red dashed line. d. duodenum; p. pancreas; s. spleen; stomach;. Immunofluorescence for exocrine markers amylase and mucin reveals normal exocrine formation in the pancreatic remnant of *Rfx6-Cre; Ctnnb1*^*tm1Mmt/+*^ mice (C, D). Decreased endocrine structures in *Rfx6-Cre; Ctnnb1*^*tm1Mmt/+*^ mice (F) compared to control pancreata (E). Higher magnification pictures are shown in insets. Reduced epithelium in early embryonic *Rfx6-Cre; Ctnnb1*^*tm1Mmt/+*^ pancreata compared to control pancreata, as revealed by carboxypeptidase A1 (G, H) and Pdx-1 (I, J) immunohistochemistry. (G) Quantification of Cpa1-positive area in *Rfx6-Cre; Ctnnb1*^*tm1Mmt/+*^ and control E13.5 embryonic pancreas. Scale bars, 100 μm.
(TIF)

S4 Fig Loss of pancreatic tissue in *Rfx6-Cre; Ctnnb1*^*tm1Mmt/+*^ embryos. Representative sequential paraffin sections stained for Pdx-1 antibody of *Rfx6-Cre; Ctnnb1*^*tm1Mmt/+*^ (A-F) and control (G-L) E13.5 embryonic pancreas. d, duodenum; dp, dorsal pancreas; li, liver; p, pancreas; s, spleen; st, stomach; vp, ventral pancreas.
(TIF)

S5 Fig Gastric and intestinal markers in *Rfx6-Cre; Ctnnb1*^*tm1Mmt/+*^ embryonic pancreata. Low magnification pictures of *Rfx6-Cre; Ctnnb1*^*tm1Mmt/+*^ (D, E, F) and control (A, B, C) E18.5 embryonic pancreas stained for Sox2, Cdx2 and β-catenin. Higher magnification pictures of these images are shown in Fig 4. d, duodenum; dp, dorsal pancreas; li, liver; p, pancreas; s, spleen; st, stomach; vp, ventral pancreas.
(TIF)

S1 Table Primary antibodies. (DOCX)

S2 Table Primer sequences. (DOCX)
