# Supplementary figures and images for: Loss of Pancreas upon Activated Wnt Signaling Is Concomitant with Emergence of Gastrointestinal Identity
Source: PLoS One. 2016 Oct 13;11(10):e0164714. doi: 10.1371/journal.pone.0164714 (PMC5063371; doi:10.1371/journal.pone.0164714)

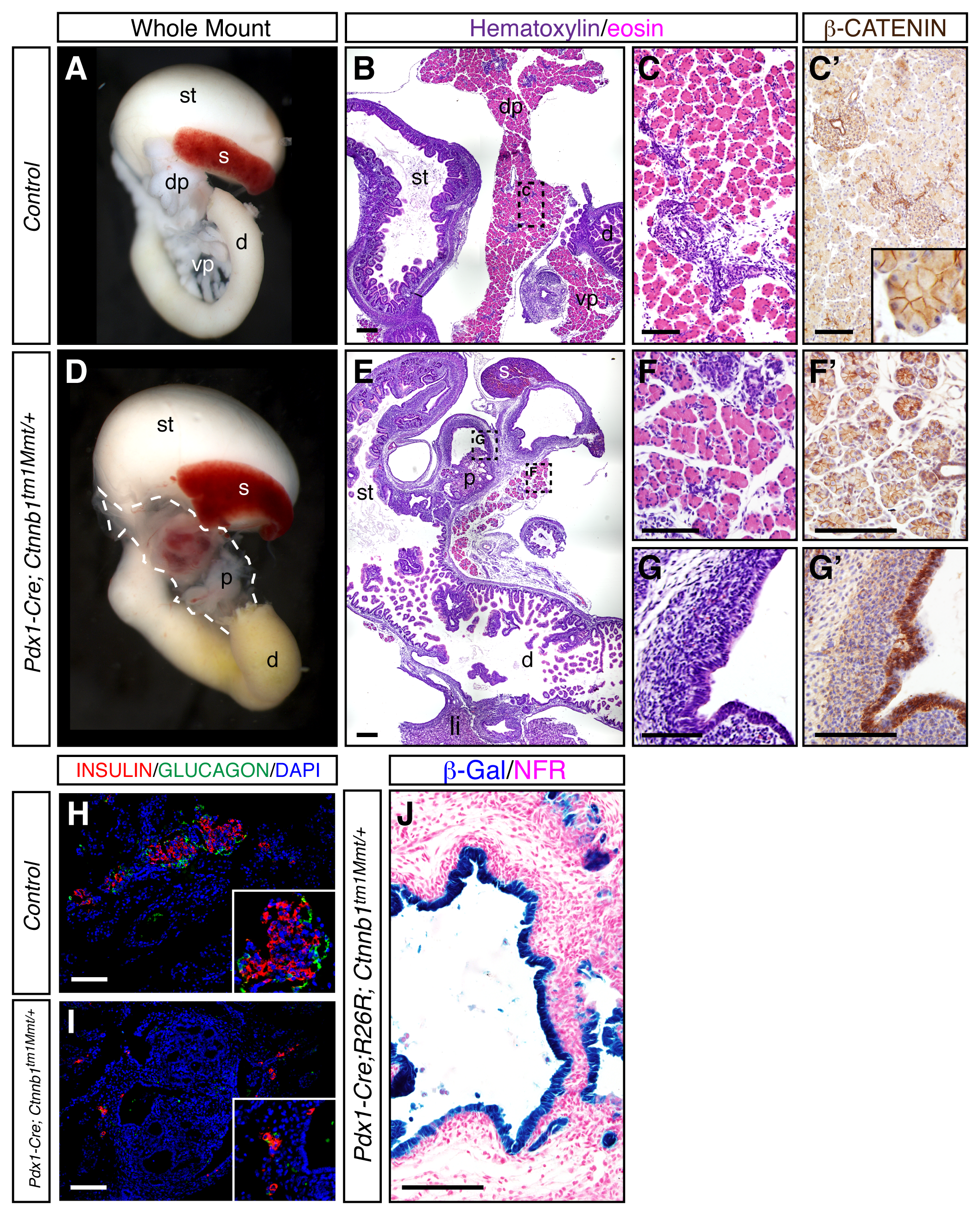

Supplement: S1 Fig — (A, D) Whole mount pictures of representative P0 pancreata from Pdx1-Cre; Ctnnb1tm1Mmt/+ and control embryos. Low magnification pictures of Hematoxylin/eosin-stained paraffin sections of control (B) and Pdx1-Cre; Ctnnb1tm1Mmt/+ P0 mice (E). The boxed areas in B and E are shown at higher magnification in C, F and G. High magnification picture of control pancreas (C). High magnification picture of the scarce normal pancreatic tissue (F) and pancreatic cysts (G) in Pdx1-Cre; Ctnnb1tm1Mmt/+ mice. Immunohistochemistry reveals membranous localization of β-catenin in control pancreatic tissue (C') and in normal pancreatic tissue of Pdx1-Cre; Ctnnb1tm1Mmt/+ mice (F'). Nuclear β-catenin localization is found in epithelial cells of pancreatic cystic structures of Pdx1-Cre; Ctnnb1tm1Mmt/+ P0 mice (G'). Reduced islet formation Pdx1-Cre; Ctnnb1tm1Mmt/+ newborn mice (H) compared to control mice (I). Higher magnification pictures are shown in insets. (J) Genetic labeling of pancreatic cells by detection of reporter β-galactosidase activity in newborn P0 Pdx1-Cre; R26R; Ctnnb1tm1Mmt/+ pancreata. β-galactosidase was restricted to the epithelial compartment. Note that the epithelial cells lining the cysts were positive for β-galactosidase. Scale bars, 100 μm. d, duodenum; dp, dorsal pancreas; Li, liver st, stomach; vp, ventral pancreas. (TIF) [file pone.0164714.s001.tif]

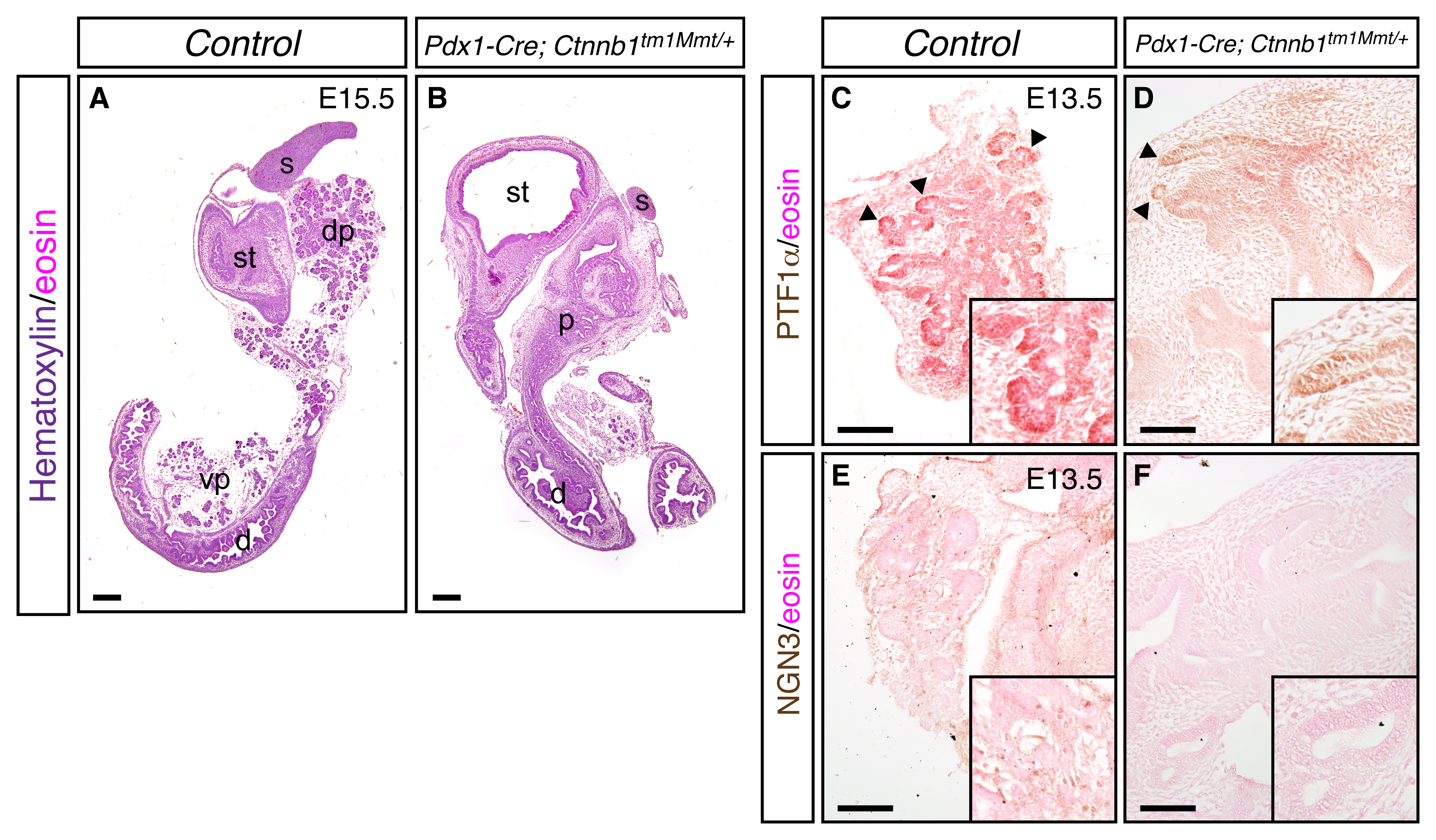

Supplement: S2 Fig — Hematoxylin/eosin-stained paraffin sections of E15.5 control (A) and Pdx1-Cre; Ctnnb1tm1Mmt/+ pancreata (B). (D) Reduced PTF1a expression in E13.5 Pdx1-Cre; Ctnnb1tm1Mmt/+ embryonic pancreata compared to control embryos (C). (F) Reduced NGN3 expression in E13.5 Pdx1-Cre; Ctnnb1tm1Mmt/+ embryonic pancreata compared to control embryos (E). Higher magnification pictures are shown in insets. d, duodenum; dp, dorsal pancreas; p, pancreas; s, spleen; st, stomach; vp, ventral pancreas. (TIF) [file pone.0164714.s002.tif]

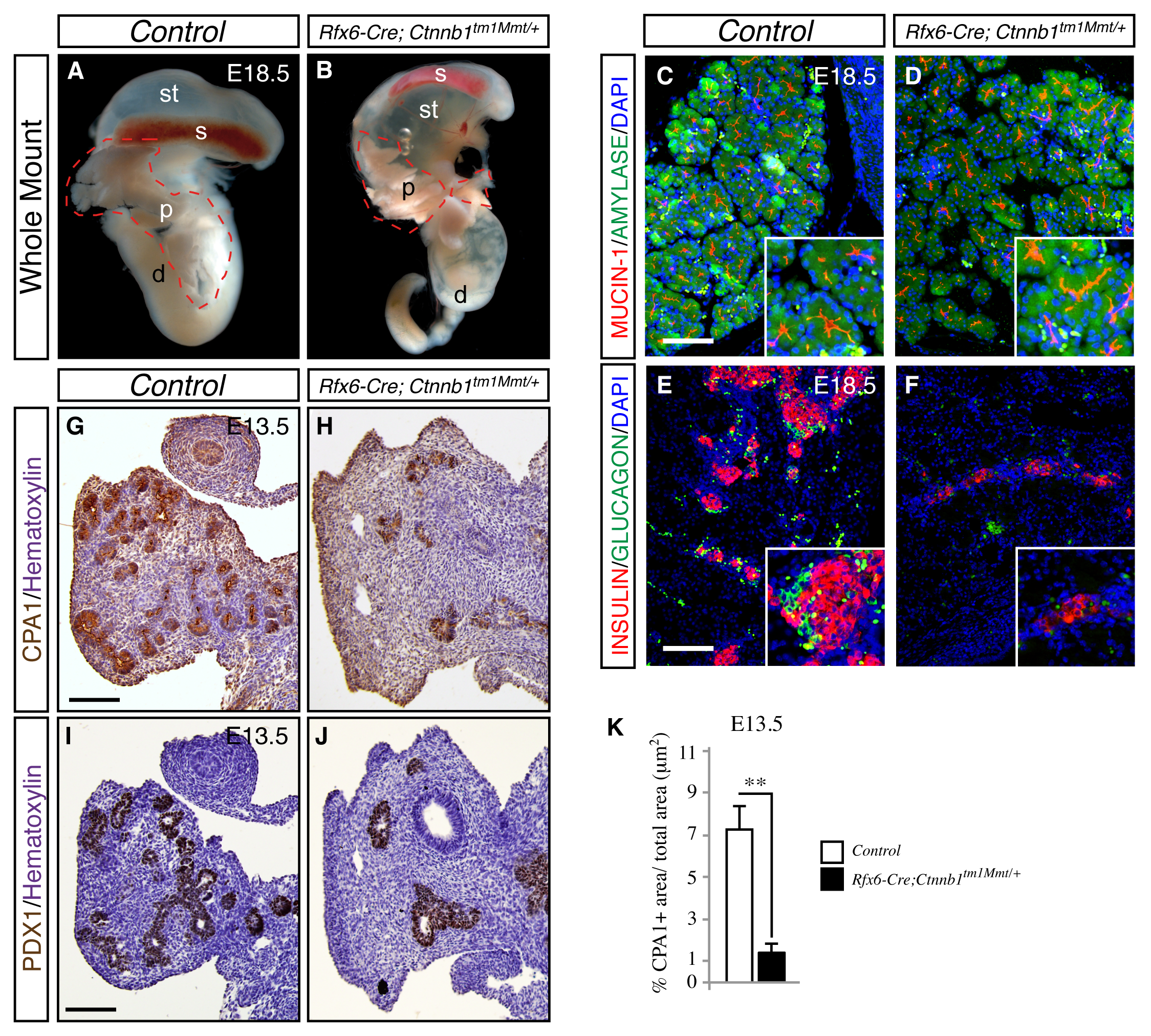

Supplement: S3 Fig — Whole mount pictures of representative P0 pancreata from Rfx-Cre; Ctnnb1tm1Mmt/+ (A) and control mice (B). Pancreatic tissue is outline in red dashed line. d. duodenum; p. pancreas; s. spleen; stomach;. Immunofluorescence for exocrine markers amylase and mucin reveals normal exocrine formation in the pancreatic remnant of Rfx6-Cre; Ctnnb1tm1Mmt/+ mice (C, D). Decreased endocrine structures in Rfx6-Cre; Ctnnb1tm1Mmt/+ mice (F) compared to control pancreata (E). Higher magnification pictures are shown in insets. Reduced epithelium in early embryonic Rfx6-Cre; Ctnnb1tm1Mmt/+ pancreata compared to control pancreata, as revealed by carboxypeptidase A1 (G, H) and Pdx-1 (I, J) immunohistochemistry. (G) Quantification of Cpa1-positive area in Rfx6-Cre; Ctnnb1tm1Mmt/+ and control E13.5 embryonic pancreas. Scale bars, 100 μm. (TIF) [file pone.0164714.s003.tif]

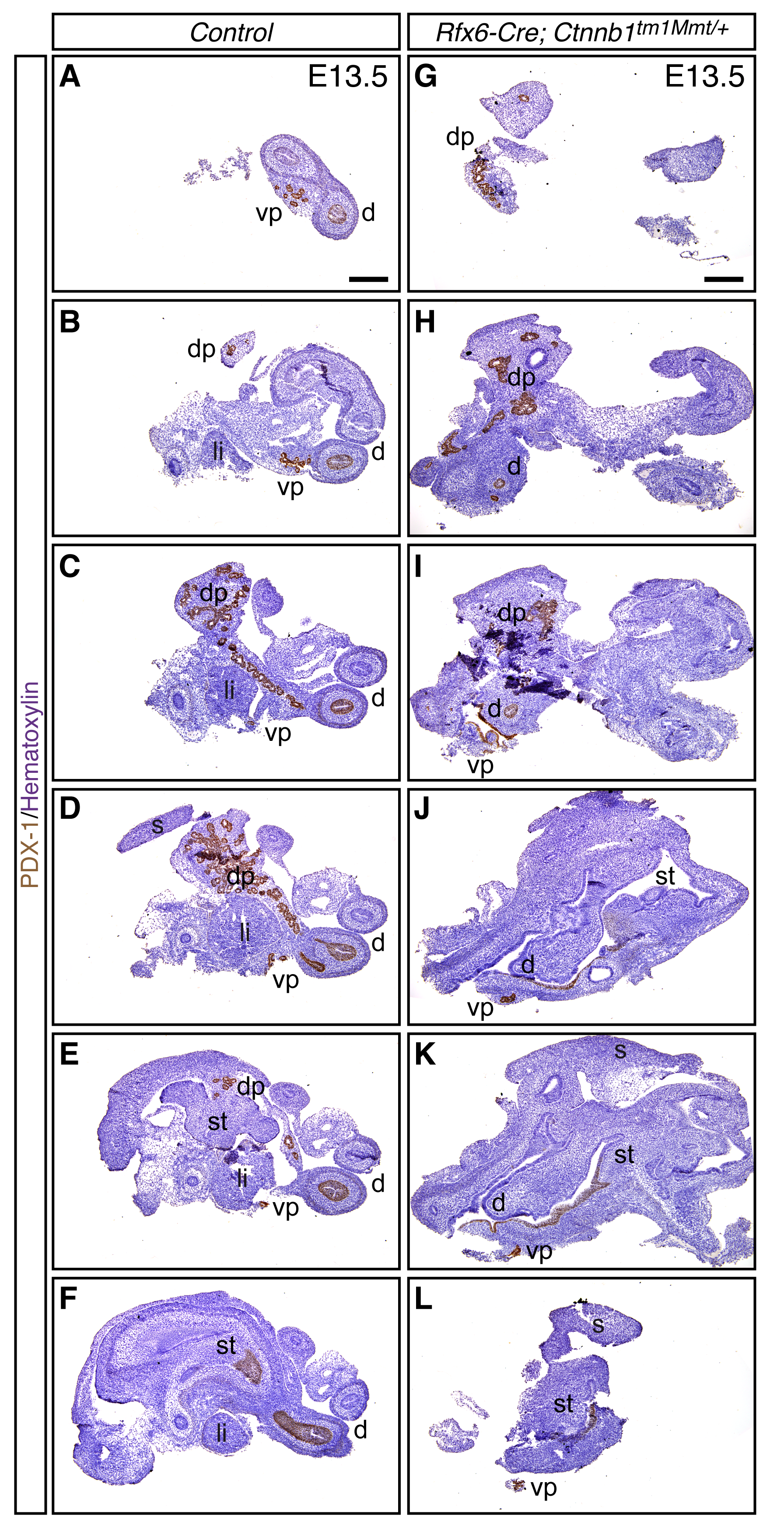

Supplement: S4 Fig — Representative sequential paraffin sections stained for Pdx-1 antibody of Rfx6-Cre; Ctnnb1tm1Mmt/+ (A-F) and control (G-L) E13.5 embryonic pancreas. d, duodenum; dp, dorsal pancreas; li, liver; p, pancreas; s, spleen; st, stomach; vp, ventral pancreas. (TIF) [file pone.0164714.s004.tif]

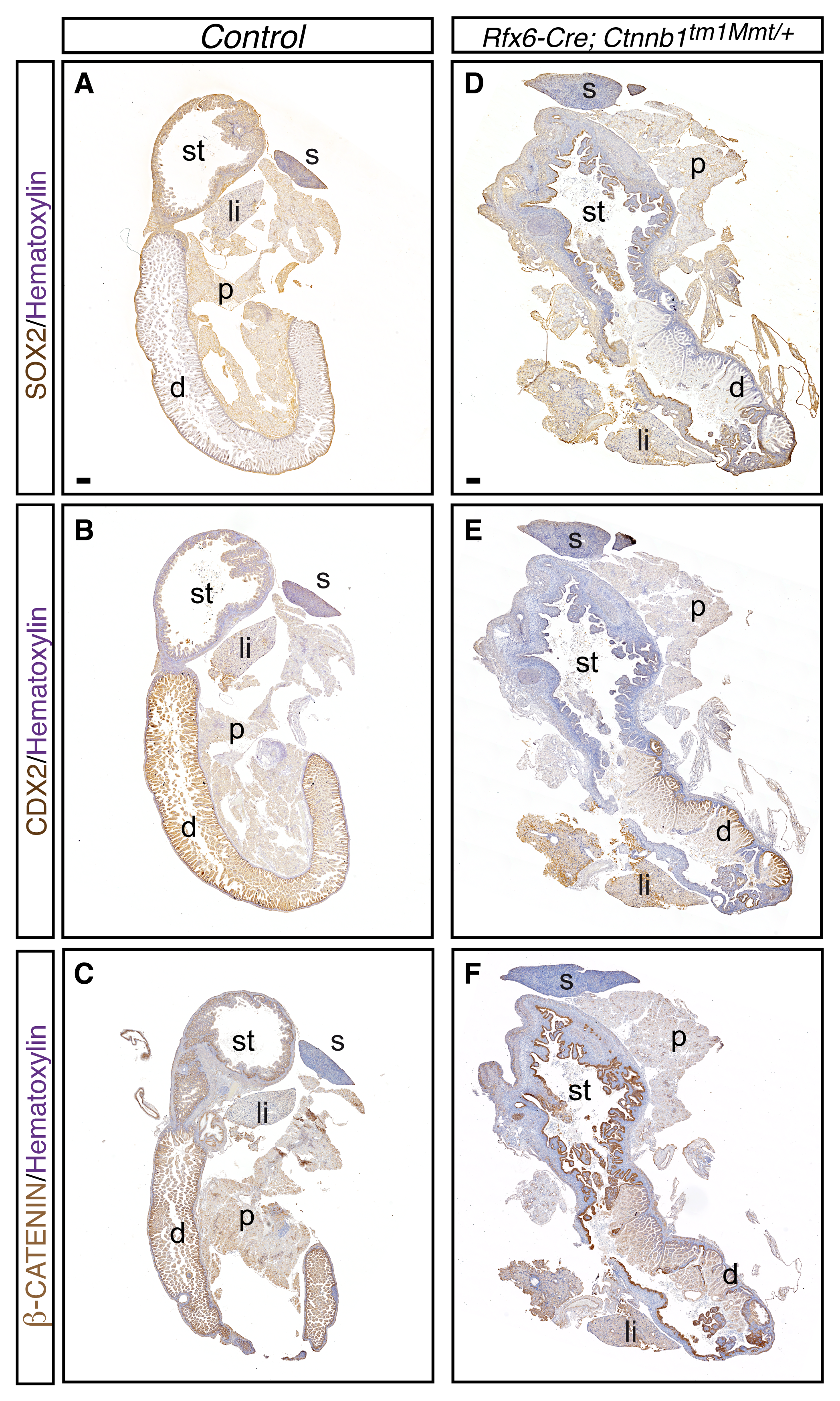

Supplement: S5 Fig — Low magnification pictures of Rfx6-Cre; Ctnnb1tm1Mmt/+ (D, E, F) and control (A, B, C) E18.5 embryonic pancreas stained for Sox2, Cdx2 and β-catenin. Higher magnification pictures of these images are shown in Fig 4. d, duodenum; dp, dorsal pancreas; li, liver; p, pancreas; s, spleen; st, stomach; vp, ventral pancreas. (TIF) [file pone.0164714.s005.tif]
