# Supplementary material for: Loss of Pancreas upon Activated Wnt Signaling Is Concomitant with Emergence of Gastrointestinal Identity
Source: PLoS One. 2016 Oct 13;11(10):e0164714. doi: 10.1371/journal.pone.0164714 (PMC5063371; doi:10.1371/journal.pone.0164714)
Supplement: S1 Table — (DOCX) [file pone.0164714.s006.docx]

**S1 Table. Primary antibodies**

| Antibody | Species | Dilution | Source (ref.) |
| --- | --- | --- | --- |
| Anti-α-AMYLASE | Rabbit | 1:300 | Sigma (A8273) |
| Anti-β-CATENIN | Mouse | 1:200 | BD Transduction (610153) |
| Anti-CDX2 | Rabbit | 1:1000 | Bethyl (A300-691A) |
| Anti-CPA1 | Rabbit | 1:1000 | AbD (1810-0006) |
| Anti-E-CADHERIN | Mouse | 1:200 | BD Transduction (610181) |
| Anti-FOXA2 | Rabbit | 1:200 | Millipore (07-633) |
| Anti-KI67 | Rabbit | 1:200 | Thermo Scientific (RM9106-S0) |
| Anti-LAMININ | Rabbit | 1:100 | Sigma (L9393) |
| Anti-MUCIN1 | Hamster | 1:200 | Thermo Scientific (HM1630) |
| Anti-MUCIN2(H300) | Rabbit | 1:500 | Santa Cruz (15334) |
| Anti-MUCIN5AC | Mouse | 1:200 | Thermo Scientific (MS-145-P1) |
| Anti-NKX2.2 | Mouse | 1:200 | Hybridoma Bank (74.5ª5) |
| Anti-NKX6.1 | Mouse | 1:200 | Hybridoma Bank (F55A10) |
| Anti-PDX1 | Guinea Pig | 1:200 | Abcam (ab7308) |
| Anti-PDX1 | Mouse | 1:100 | Hybridoma Bank (F6A11) |
| Anti-SHH | Goat | 1:50 | R&D (AF445) |
| Anti-SMA | Mouse | 1:500 | Sigma (A2547) |
| Anti-SOX2 | Rabbit | 1:2000 | Millipore (AB5603) |
| Anti-TUJ1 | Rabbit | 1:1000 | Abcam (ab18207) |
