# Supplementary material for: Loss of Pancreas upon Activated Wnt Signaling Is Concomitant with Emergence of Gastrointestinal Identity
Source: PLoS One. 2016 Oct 13;11(10):e0164714. doi: 10.1371/journal.pone.0164714 (PMC5063371; doi:10.1371/journal.pone.0164714)
Supplement: S2 Table — (DOCX) [file pone.0164714.s007.docx]

**S2 Table. Primer sequences**

| **Primer Name** | **Oligo Sequence** | **Reference/ PrimerBankID** |
| --- | --- | --- |
| Axin2-Fwd | CTCCCCACCTTGAATGAAGA | Li et al., 2011 |
| Axin2-Rv | ACTGGGTCGCTTCTCTTGAA |  |
| Barx1-Fwd | AAGACGTGGTATCAAAATCGGAG |  |
| Barx1-Rv | GCCACCTTGCAGCACTATT |  |
| Cdx2-Fwd | GCGACAAGGGCTTGTTTAGA |  |
| Cdx2-Rv | AAGGCTTGTTTGGCTCGTTA |  |
| CyclinD2-Fwd | GCTTGTAGAGGCTGCTTTGGTT | Rieck et al., 2009 |
| CyclinD2-Rv | AGAGGAGTCCCGTGTCAGTAGG |  |
| CycloA-Fwd | TCACAGAATTATTCCAGGATTCATG | Puri et al., 2009 |
| CycloA-Rv | TGCCGCCAGTGCCATT |  |
| Dkk2-Fwd | TCGCCACTTCTGGACCAAAA | Pasca di Magliano M et al., 2007 |
| Dkk2-Rv | TGCACAGTCACACCTCTGGAAA |  |
| Dkk3-Fwd | CGCTGTTGCTAGAAACGCTGT | Pasca di Magliano M et al., 2007 |
| Dkk3-Rv | TCCCCCAAATCCATCATGAG |  |
| Foxf1-Fwd | ACGCCGTTTACTCCAGCTC | 6753902a1 |
| Foxf1-Rv | CGTTGTGACTGTTTTGGTGAAG |  |
| FoxL1-Fwd | TGCCGCATTCCACAGCATAGTC | Sackett et al., 2009 |
| FoxL1-Rv | CAAAGTGAGTTCCAGGACAGCCAG |  |
| Gli1-Fwd | GCCACACAAGTGCACGTTTG | Lau & Hebrok, 2010 |
| Gli1-Rv | AAGGTGCGTCTTGAGGTTTTCA |  |
| Hhex-Fwd | CGGACGGTGAACGACTACAC | 6680219a1 |
| Hhex-Rv | CGTTGGAGAACCTCACTTGAC |  |
| Lef1-Fwd | AGTGCAGCTATCAACCAGATCCT | Pasca di Magliano M et al., 2007 |
| Lef1-Rv | TTTCCGTGCTAGTTCATAGTATTTGG |  |
| Lgr5-Fwd | CTGCCCATCACACTGTCACT | Stecca & Altaba, 2009 |
| Lgr5-Rv | GCAGAGGCGATGTAGGAGAC |  |
| Myocd-Fwd | AGGAAGTTCCGATCAGTCTTACA | 154240725c1 |
| Myocd-Rv | GGTATTAAGCCTTGGTTAGCCAG |  |
| Nkx2.2-Fwd | CGGGCGGAGAAAGCATT | Carrasco et al., 2012 |
| Nkx2.2-Rv | TCCACCTTGCGGACACTATG |  |
| Nkx6.1-Fwd | ACGCTTGGCCTATTCTCTGG | Cano et al., 2008 |
| Nkx6.1-Rv | CGTGCTTCTTTCTCCACTTGGT |  |
| Pdx-1-Fwd | CTTAACCTAGGCGTCGCACAA | Carrasco et al., 2012 |
| Pdx-1-Rv | GAAGCTCAGGGCTGTTTTTCC |  |
| Pitx1-Fwd | CTGCCGGCTACTCCTACAAC | Kim et al., 2011 |
| Pitx1-Rv | GGCATGGTCATGGAAGAGAT |  |
| Ptch-Fwd | CCCTAACAAAAATTCAACCAAACCT | Lau & Hebrok, 2010 |
| Ptch-Rv | GCATATACTTCCTGGATAAACCTTGAC |  |
| Shh-Fwd | GCAGGTTTCGACTGGGTCTA | Stecca & Altaba, 2009 |
| Shh-Rv | GAAGGTGAGGAAGTCGCTGT |  |
| Sox2-Fwd | GCGGAGTGGAAACTTTTGTCC | 31543759a1 |
| Sox2-Rv | CGGGAAGCGTGTACTTATCCTT |  |
| Sox9-Fwd | GCAGACCAGTACCCGCATCT | Carrasco et al., 2012 |
| Sox9-Rv | TTCAGCAGCCTCCAGAGCTT |  |
